# Fast, vacancy-free climb of prismatic dislocation loops in bcc metals

**DOI:** 10.1038/srep30596

**Published:** 2016-08-23

**Authors:** Thomas D. Swinburne, Kazuto Arakawa, Hirotaro Mori, Hidehiro Yasuda, Minoru Isshiki, Kouji Mimura, Masahito Uchikoshi, Sergei L. Dudarev

**Affiliations:** 1 CCFE, Culham Science Centre, Abingdon, Oxon, OX14 3DB, UK; 2 Department of Materials Science, Faculty of Science and Engineering, Shimane University, 1060 Nishikawatsu, Matsue 690-8504, Japan; 3 Research Center for Ultra-High Voltage Electron Microscopy, Osaka University, 7-1 Mihogaoka, Ibaraki, Osaka 567-0047, Japan; 4 Institute of Multidisciplinary Research for Advanced Materials, Tohoku University, 2-2-1 Katahira, Aoba-ku, Sendai 980-8577, Japan

## Abstract

Vacancy-mediated climb models cannot account for the fast, direct coalescence of dislocation loops seen experimentally. An alternative mechanism, self climb, allows prismatic dislocation loops to move away from their glide surface via pipe diffusion around the loop perimeter, independent of any vacancy atmosphere. Despite the known importance of self climb, theoretical models require a typically unknown activation energy, hindering implementation in materials modeling. Here, extensive molecular statics calculations of pipe diffusion processes around irregular prismatic loops are used to map the energy landscape for self climb in iron and tungsten, finding a simple, material independent energy model after normalizing by the vacancy migration barrier. Kinetic Monte Carlo simulations yield a self climb activation energy of 2 (2.5) times the vacancy migration barrier for 1/2〈111〉 (〈100〉) dislocation loops. Dislocation dynamics simulations allowing self climb and glide show quantitative agreement with transmission electron microscopy observations of climbing prismatic loops in iron and tungsten, confirming that this novel form of vacancy-free climb is many orders of magnitude faster than what is predicted by traditional climb models. Self climb significantly influences the coarsening rate of defect networks, with important implications for post-irradiation annealing.

Dislocation glide dominates plastic flow at low homologous temperatures, but confinement to a glide surface significantly restricts the evolution of a defect network^1^. Dislocation climb, which requires concurrent mass transport, is typically much slower than glide but allows migration off the glide surface, giving rise to a wide range of important plasticity mechanisms including network coarsening^2^,^3^ and creep^4^. Climb is known^5^,^6^,^7^,^8^,^9^ to be particularly important in post-irradiation annealing, accelerating the coalescence of the many small self interstitial atom (SIA) and vacancy clusters produced in a cascade event, increasing the characteristic length scale of the dislocation network and transforming the mechanical response.

The most widely studied atomic mechanism for climb transports mass through a pre-existing vacancy atmosphere, here referred to as vacancy mediated climb (VMC), with dislocations acting as perfect sources and sinks for vacancies^2^,^3^,^4^. By emitting and absorbing vacancies, dislocation segments can move off their glide plane; as the self stress of a dislocation loop is approximately proportional to the inverse of the loop radius^1^, larger loops tend to grow at the expense of smaller loops whilst maintaining the equilibrium vacancy concentration, a feature that is clearly observed in some experiments^7^. As the vacancy current at a dislocation is proportional to the product of the vacancy concentration and diffusivity 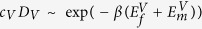, the rate of VMC is controlled by the large activation energy 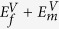 for vacancy formation and migration^3^, meaning VMC mechanisms are only expected to be active at high homologous temperatures^4^.

However, it has long been recognized^5^,^6^,^7^,^9^,^10^ that prismatic loops can also migrate away from their glide cylinder, with no observable change in size, driving loop coalescence. The climb motion in this case is clearly not VMC as the loop area is conserved; furthermore, we confirm previous findings^5^,^9^,^11^ that the climb velocities are many orders of magnitude larger than velocities predicted by VMC models and active at much lower temperatures. An alternative ‘self climb’ model was proposed to account for these puzzling observations^5^,^10^,^12^,^13^,^14^, where loops are able to migrate in their habit plane due to the diffusion of self interstitial atoms (SIA) around the loop perimeter, in close analogy to the diffusion mechanism of large adatom islands^15^. Theoretical analyses^5^,^12^,^13^,^14^ (see below) of self climb found a mobility law of


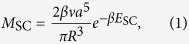


where *R* is the loop radius, a is the lattice parameter, *β* = 1/k_B_*T, ν* is an attempt frequency and *E*_SC_ is a characteristic activation energy. As no vacancy atmosphere is required, self climb is suspected to be much faster than VMC as a factor 
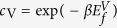
 is absent, but this cannot be confirmed without a quantitative calculation of *E*_SC_, the critical, rate controlling parameter in (1). In this paper, we use the results of over a hundred molecular statics barrier climbing calculations and kinetic Monte Carlo (KMC) simulations to explore the energy landscape for pipe diffusion around SIA loops. The pipe diffusion process in self climb effectively enables non-glide structural fluctuations of the loop, which enables thermally activated diffusive climb motion, with a characteristic activation energy *E*_SC_. Our main result is that the activation energy for self climb is given by


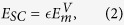


where 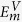 is the *vacancy* migration barrier and 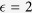 or 2.5 for 〈111〉 or 〈100〉 loops. To complete the (1), *ν* is taken as the attempt frequency for vacancy diffusion^16^,^17^. We find that the self climb activation energy 
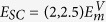
 is much lower than the effective activation energy of 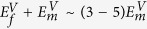 for VMC in bcc metals^18^, resulting in much larger self climb velocities. In fcc metals 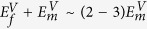
19, implying that self climb and VMC velocities may be of comparable magnitudes, a question we leave for a future investigation. Our observed activation energy is consistent with experimental estimates of *E*_SC_ that find *E*_SC_ ~ (0.4–0.7) *E*_f+Em_ over a wide range of materials^5^,^12^,^13^,^14^.

The fully parametrized mobility law is tested against direct *in situ* transmission electron microscopy (TEM) observations of SIA loops in iron at 500 °C, where isolated SIA loops are seen to execute unbiased self climb diffusion perpendicular to their Burgers vector, whilst adjacent SIA loops directly coalesce through self climb, driven by elastic interaction forces. We also analyze recently published observations of SIA loop coalescence in tungsten^9^. In all cases, we find the climb diffusivities and coalescence times measured experimentally are around six orders of magnitude faster than those predicted by the existing VMC models but in very good agreement with dislocation dynamics simulations that allow self climb and glide. The influence of self climb in bcc metals is further demonstrated through simulations of prismatic loop coarsening, which we compare to an analytical model for coarsening rates found from VMC simulations^3^.

## Results

### Energy landscape for pipe diffusion in SIA loops

**Figure 1:**
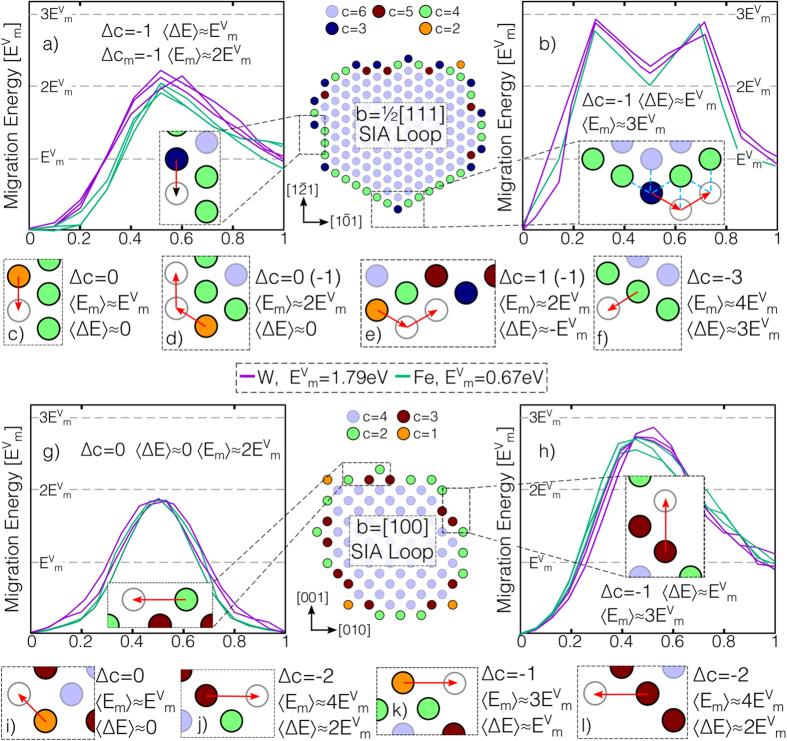
A representative sample of energy barriers for pipe diffusion around a large number of irregular 1/2〈111〉 and 〈100〉 prismatic loops in iron and tungsten, two of which are shown. Defining a ‘bond’ as a nearest SIA neighbor, we find a clear correlation between the bond number and the expected migration barrier and total energy change, which can account for all observed pathways when normalized by the vacancy migration barrier (see main text for details).

As self climb is driven by pipe diffusion around the perimeter of SIA loops, we have simulated a large number (>100) of possible diffusion pathways for 1/2〈111〉 and 〈100〉 SIA loops in iron and tungsten (see methods). It was found that all relaxed pathways between metastable states could be approximated as *a*/2〈111〉 jumps for 1/2〈111〉 loops, and *a*/2〈111〉 or *a*〈100〉 jumps for 〈100〉 loops. As shown in Fig. 1, after normalizing by the vacancy migration energy 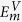 for the interatomic potential, we find that the total change in energy Δ*E* and migration barrier *E*_*m*_ for a jumping atom can be accurately modeled simply by counting the number *c* of nearest SIA neighbors along the path, i.e. for each point on the path, 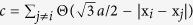, where Θ(*x*) is the Heaviside step function. For SIAs in the interior of an 1/2〈111〉 loops *c* = 6, whilst for 〈100〉 loops *c* = 4; classifying the SIAs on a loop perimeter in this manner allowed us to produce the distribution of migration pathways shown in Fig. 1a,b,f,g. Collating the results of our NEB calculations revealed the approximate relationship


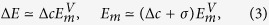


where Δ*c* is the change in *c* between the initial and final configuration, *σ* = 1 for jumps along *a*/2〈111〉 (c.f. Fig. 1c,i) and *σ* = 2 for jumps along *a*/2〈100〉 (c.f. Fig. 1g). The remarkably simple relationship (3) can be rationalized by noting that the ‘hopping’ of a single SIA is akin to the vacancy migration process; in a first approximation, vacancy migration in the bulk is a hop of single atom, which requires ‘breaking’ a single nearest neighbors bond. A SIA on the perimeter of a prismatic loop has *c* additional bonds compared to an atom in the bulk, of which it must ‘break’ but one in order to jump along *a*/2〈111〉 and two to jump along *a*〈100〉, in close analogy to the vacancy migration mechanism. In contrast, the SIA migration barrier is typically much lower than 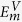 in bcc metals^18^ due to the extended strain field of SIA defects, meaning that no ‘hopping’ occurs under SIA migration^20^. Generally, variations in the observed total energy change Δ*E* and migration barrier *E*_*m*_ were not observed to be greater than 15% from those predicted by (3), which typically overestimates *E*_*m*_, meaning (3) will give an lower bound on the self climb mobility.

As self climb is known to play an important role in prismatic loop coalescence, we also determined the effect of a nearby loop’s elastic field on the observed energy barriers by performing the same NEB calculations in the presence of another prismatic loop, rotating/inverting the loop of interest by a bcc symmetry operation (in *O*_*h*_) to vary the elastic environment of the jumping atom. For loop separations of more than ~5*a* we found variations in the energy barrier of less than 5%. As such close proximity was needed to produce observable changes we expect the self-climb energy landscape to be essentially unaffected by any surrounding defect network, even line dislocations which are not elastically neutral. The goal of the NEB calculations is to parametrize KMC simulations; an alternative approach to the simple model (3) used here would be to correlate all observed migration pathways for a given material with some detailed structural signature for the jumping atom involving second and third nearest neighbors, which could then be used to construct a large table of transition rates for use in KMC^21^. However, we believe our simple bond counting model (3), tested in iron and tungsten, provides a high degree of quantitative accuracy (given the expected tolerances of the interatomic potential) coupled with an attractive simplicity, resulting in a universal model for self climb in bcc metals and allowing the treatment of large, experimentally observable SIA loops.

### Self climb mobility

**Figure 2:**
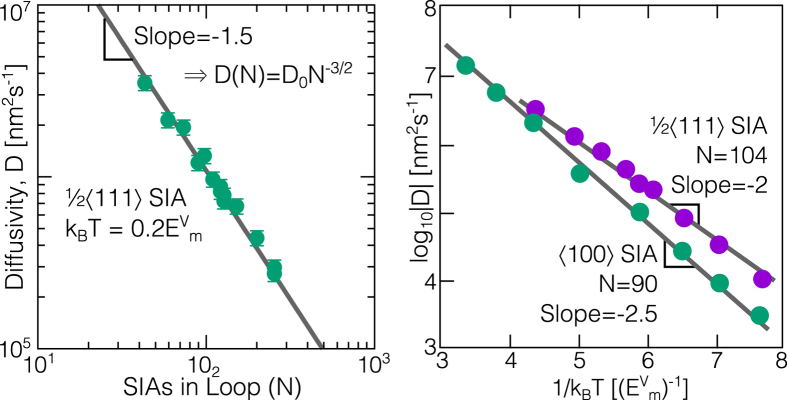
Results from KMC simulations of SIA loop self climb. Left: log-log plot showing the *D*_SC_ ∝ *N*^−3/2^ relationship predicted by (4). Right: Arrhenius plots for 1/2〈111〉 and 〈100〉 SIA loops, giving clear activation energies of 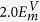 and 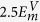.

As the presence of a realistic external stress field (due to a nearby dislocation loop) was found to have only a weak influence over the measured pipe diffusion migration barriers, we can calculate the self climb velocity **v**_cl_ under a climb force **F**_cl_ in the linear response regime **v**_cl_ = *M*_SC_**F**_cl_, where the self climb mobility is given by the Einstein relation *M*_SC_ = *βD*_SC_. We therefore only need to calculate the self climb diffusivity *D*_SC_ of an isolated loop under no applied stress, obviating the need to include computationally demanding long range elastic interactions in KMC^22^. In the presence of multiple migration pathways, the effective activation energy *E*_SC_ will reflect the rate limiting process for loop migration^15^; as shown in Fig. 2, our KMC simulations reveal (see methods) an effective activation energy 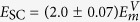 for 1/2〈111〉 SIA loops and 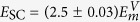 for 〈100〉 SIA loops, comparable to the energy barrier required for a lone SIA to traverse a corner or step on the loop perimeter (c.f. Fig. 1d,k). The larger activation energy for 〈100〉 self climb can be attributed to the larger energy barrier for SIA migration along 〈100〉 directions. We note that in both cases the self-climb activation energy is much lower than that required for the ‘nucleation’ of lone SIAs from flat low index directions (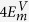 for both SIA loops, c.f. Fig. 1f,l). Simulations where such nucleation processes are explicitly forbidden exhibit unchanged diffusivities, confirming that migrating SIAs are produced around corners or roughened structures on the loop perimeter, emphasizing the importance of considering ‘non-perfect’ defect structures when modeling self climb. We note that these imperfect defect structures could also be created through VMC. To derive an analytical expression for the self climb diffusivity *D*_SC_, consider an isolated loop of *N* SIAs with an approximately circular shape, such that 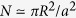. The center of mass diffusivity is given by *D*_SC_ = ∑_*ij*_*D*_*ij*_/*N*^2^, where 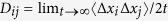. As only 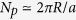 SIAs on the perimeter are able to move (*D*_*ij*_ ≠ 0), under the assumption of uncorrelated perimeter atoms we obtain


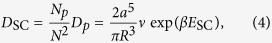


which with the Einstein relation *M*_SC_ = *βD*_SC_ gives equation (1) for the self climb mobility. The predicted relationship *D*_SC_ ∝ *R*^−3^ ~ *N*^−3/2^ is in agreement with our simulations (Fig. 2) and similar analysis of perimeter diffusion controlled transport of adatom islands^15^,^23^.

### Comparison to TEM observations of self climb

**Figure 3:**
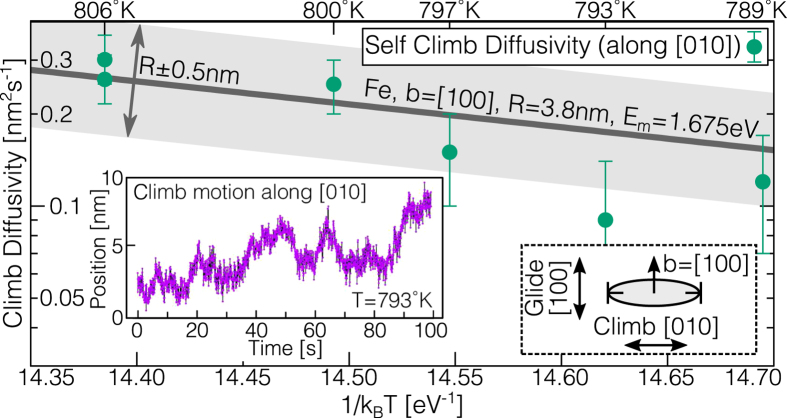
Self climb diffusivity of an 〈100〉 SIA loop in Fe. The trajectory (observed as a projection on (001) (inset) clearly shows a climb component along [010], perpendicular to the Burgers vector *b* = *a*[100]. The predicted self climb diffusivity (4), with vacancy attempt frequency taken from DFT calculations^16^ shows good agreement.

**Figure 4:**
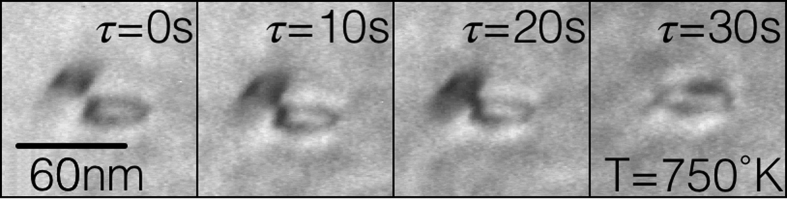
TEM observation of two 1/2〈111〉 prismatic loops in iron at 750 K, coalescing over a time period of 30 s. This processes corresponds to the first entry in Table 1.

**Figure 5:**
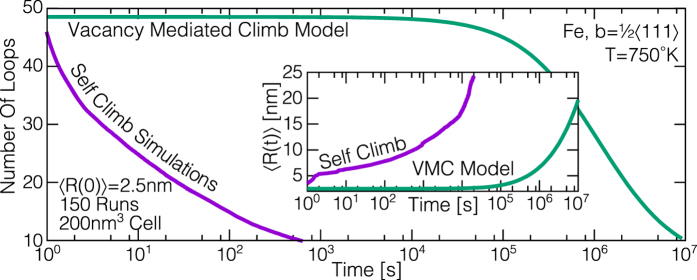
Self climb enabled dislocation dynamics simulations of SIA loop coarsening, using parameters appropriate for iron at 750 K. Our results are compared to an analytical model of VMC loop coarsening, which is in very good agreement with VMC enabled DD simulations^3^. We see that self climb coarsening rates are three to four orders of magnitude greater than those predicted by VMC.

To validate our self-climb mobility (1) and diffusivity (4), transmission electron microscopy (TEM) was used to observe the dynamics of both isolated and interacting SIA loops in ultra-high purity *α*-Fe (RRR: 7900) and high-purity *α*-Fe (RRR: 2000, purity: 99.998 wt.%), respectively. An isolated *b* = *a*[100] SIA loop of radius 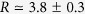 nm was observed freely diffusing both along *and* perpendicular to its Burgers vector of [100]. We identify the perpendicular motion along [010] as a one dimensional projection of self climb in (100) planes. Through analysis of the mean squared displacement along [010] (Fig. 3, inset) self climb diffusivities were extracted at five temperatures, from 793 K to 806 K, well below temperatures where vacancy-mediated climb is expected to be active. Figure 3 shows that equation (4), with parameters for a 〈100〉 SIA loop of radius 3.8 nm in iron, shows excellent agreement with the experimentally determined climb diffusivity, agreeing to within 10% apart from the measurement at 793 K. As a further validation of our model we implemented the self climb mobility law (1) in simple dislocation dynamics (DD) simulations that allow glide and self climb along with isotropic elastic interactions (see methods), in order to simulate a variety of loop coalescence processes seen under the TEM. A representative observation is show in Fig. 4. For comparison, we also replaced the self-climb mobility *M*_scl_ law by an approximate VMC mobility (Equation 10 in ref. 3)


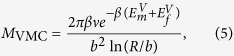


to give a measure of the expected coalescence times if VMC was the only active climb mechanism. Using the full-field elastic interaction energy given by Mura’s formula^1^, we investigated the various binary loop coalescence processes that were seen in our experiments and similar observations from other TEM studies in iron^24^ and tungsten^9^. The DD simulations did not account for the presence of a free surface nor the surrounding defect network, but the shape and position of two loops, which were either circular or pill shaped (semicircles connected by two straight lines), were chosen to approximate as closely as possible those seen under the TEM. The results of these simulations are given in Table 1; despite the approximate treatment of our simulations, the calculated coalescence times are in fairly good agreement with experimental values for both iron and tungsten, whilst simulations with the VMC mobility (5) overestimate the observed coalescence times by around six orders of magnitude, highlighting both the quality of agreement with experiment and the speed of self climb compared to VMC. We emphasize that the observed non-glide motion therefore can only be accounted for by the self climb mechanisms investigated here. To further explore the influence of self climb in post-irradiation annealing, we performed similar glide/self climb DD simulations of SIA loop coarsening (see methods), using an approximate far field solution for the loop interaction, which we compare to an analytical model^7^ that has been shown to be in very good agreement with VMC-only enabled DD simulations of loop coarsening^3^. We used simulation parameters appropriate for 1/2〈111〉 SIA loops in iron at 750 K in a simulation supercell of 200 × 200 × 200 nm. An initial population of 80 loops were placed randomly in the supercell, with random radii between 1.5 nm and 3.5 nm, under the constraint that the initial average radius 〈*R*(0)〉 = 2.5 nm. An ensemble of 150 simulations were performed until less than 4 loops remained in the supercell, though the ensemble average behavior could be considered converged after approximately 50 simulations. In Fig. 5 we plot the ensemble average time evolution of the loop number *N*(*t*) and average loop radius 〈*R*(*t*)〉, along with the predictions of the analytical VMC model, 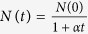 and 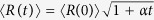, where 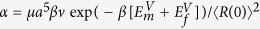 3. It is clear that the self climb coarsening rates are around three to four orders of magnitude faster than those predicted by VMC, emphasizing the necessity of including the self climb mobility law (1) into simulations of post-irradiation annealing.

## Discussion

We have derived and calculated a quantitative mobility law for self climb, by mapping out the energy landscape for pipe diffusion around a large number of randomly shaped prismatic dislocation loops in two different bcc metals which can then be explored in kinetic Monte Carlo simulations. Our results show that the activation energy for self climb is 2–2.5 times the vacancy migration barrier, which showed quantitative agreement with experimental observations of the same processes. Dislocation dynamics simulations, which account for self climb, show significantly accelerated prismatic loop coalescence rates at intermediate homologous temperatures, demonstrating that self climb is an essential component in models of microstructural evolution, even in regimes where vacancy-mediated climb can be neglected. Finally, we note that self climb activation energy *E*_SC_ is comparable to the inferred binding energy between dislocation loops and common impurities such as carbon or nitrogen (found experimentally as 1.2 eV for 1/2〈111〉 prismatic loops in iron^25^ compared to *E*_SC_ = 1.34 eV), meaning that simulations which include impurity pinning effects should also account for self climb, as the timescales for the two processes are expected to be comparable. It is also possible that self climb could play a role in defect depinning process, a topic we leave for future study.

## Methods

### Atomistic calculations

The pipe diffusion barrier calculations used the LAMMPS molecular simulation package^26^ with empirical potentials for iron by Gordon *et al*.^27^ and for tungsten by Marinica *et al*.^28^. Dislocation loops were created by inserting a random palette of SIAs on (111) or (100) planes into a perfect lattice before a structural relaxation. Away from a so called ‘magic’ number of SIAs, loops cannot form perfect hexagons or squares, resulting in a geometrically necessary roughening of the loop perimeter. Possible migration pathways were investigated by selecting at random SIAs on the perimeter of a palette, then translating the selected SIA by an *a*/2〈111〉, *a*〈100〉 or 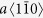 lattice vector to another perimeter position before a further relaxation. As the SIA atoms are already in off-lattice positions, a lattice vector translation also finds an off lattice position. The nudged elastic band method^29^ was then used to calculate the energy barrier between the two configurations, taking caution to choose an indexing protocol that minimizes the converged energy barrier, overcoming any ambiguity over identification of ‘the’ SIA atom in the relaxed structure of a dislocation core.

### Kinetic Monte Carlo Simulations

We used the lattice KMC code KMClib^21^ to simulate two dimensional self climb diffusion with state-state energy differences and migration barriers determined by (3), using energy units such that 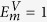, with a hexagonal or square lattice for 1/2〈111〉 or 〈100〉 loops. The simulations produce a set 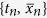 of center of mass positions 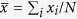. By taking the mean squared displacement at sequentially greater time delays a diffusivity was extracted using established methods^30^.

### Transmission Electron Microscopy Experiments

Thin foils with thickness less than 0.08 mm were pre-annealed at 773 K for 0.5 hours under a vacuum of 3 × 10^−6^ Pa (ultra-high purity α-Fe) and at 1073 K for 2 hours under a hydrogen atmosphere at 1 atm (high purity α-Fe) to reduce dislocation density and remove any residual stress before electro-polishing. In the TEM-visible regions, the residual-dislocation density was less than 10^9^ m^−2^, allowing the observation of individual loops with negligible effect from the stress field of residual dislocations. Loops were introduced through high-energy electron irradiation, using an ultra-high voltage TEM H-3000 by Hitachi, operated at an acceleration voltage of 2000 kV. The irradiation was performed with a beam dose of 1 × 10^25^ *e*/*m*^2^ at 290 K (ultra-high purity α-Fe) and 1 × 10^25^ *e*/*m*^2^ at 150 K (high purity α-Fe), producing SIAs that agglomerated to form loops. Behavior of SIA loops in response to specimen heating were observed using a diffraction-contrast technique at an acceleration voltage of 200 kV to avoid additional knock-on displacement, recording at a frame rate of 30 *s*^−1^ with a Gatan 676 camera.

### Dislocation Dynamics simulations

In our DD simulations, prismatic dislocation loops were allowed to glide along the direction of their Burgers vector **b** and climb in two dimensions perpendicular to their Burgers vector. The shape of the dislocation loop was fixed to be constant throughout, allowing the total elastic energy of the system *E*_elastic_, to be a function purely of the *N* loop positions (**x**_1_, **x**_2_, ... **x**_*N*_). In all our simulations the loops were assumed to have identical Burgers vectors **b**_*i*_ = **b**. The equation of motion for an individual loop *i* therefore read


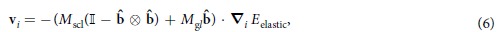


where *M*_gl_ is a glide mobility suitable for prismatic dislocation loops at high temperature^31^,^32^,^33^ and *M*_scl_ is the self climb mobility given in equation (1). For comparison, as described in the main text, we also performed identical simulations with *M*_scl_ replaced by the VMC law (5). As the glide mobility *M*_gl_ is much greater than the climb mobility *M*_scl_ we split the integration of (6), first allowing glide-only motion until a local steady state had been reached, defined as when the total magnitude of the glide force, 
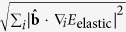
, was less than 0.02 eV/Å. At this point loops were allowed to respond to their climb forces; the loop with the largest climb force magnitude 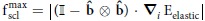 was allowed to move a distance *δr* = 1 *Å* in the direction of the climb force, and the simulation time was updated by 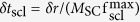. The remaining loops all migrated in the direction of their climb force a distance 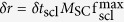.

For the binary loop coalescence simulations the elastic interaction energy was given by Mura’s formula (equation 4–40 in ref. 1) for two loops with identical Burgers vectors **b**; the loops under study were either circular or pill shaped (i.e. two semicircles connected by parallel straight lines), and were chosen to approximate as closely as possible the shape and position of the coalescing loops seen under the TEM, though the effect of the free surface and the surrounding defect network is neglected.

For the simulations treating the coarsening of a distribution of dislocation loops, all loops were taken to be collinear and circular, with coalescence occurring instantaneously on contact, whereupon the larger loop was expanded to accommodate the area of the smaller loop. To speed up the simulations we used an analytical far field solution for the loop-loop interaction^7^ for two loops of radii *R*_1_, *R*_2_ and positions **x**_1_, **x**_2_


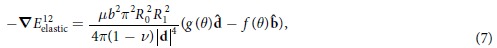


where **d** = **x**_1_ − **x**_2_, 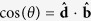, 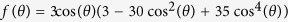 and 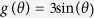 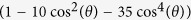. As the difference in forces across the loop is greater than that given by the far field approximation, which assumes the loop radius is negligible compared to the loop separation, the true elastic force will be greater than that in our coarsening simulations as the loops approach each other, meaning our results can be considered to provide a lower bound on the coarsening times.

## Additional Information

**How to cite this article**: Swinburne, T. D. *et al*. Fast, vacancy-free climb of prismatic dislocation loops in bcc metals. *Sci. Rep.* **6**, 30596; doi: 10.1038/srep30596 (2016).

## Figures and Tables

**Table 1:** Comparison of coalescence times of 1/2〈111〉 SIA loops from TEM observations (*τ*_ex_), DD simulations where loops can glide and self climb (*τ*_sc_) and DD simulations where loops can glide and climb via VMC^3^ (*τ*_VMC_).

	*R*_1_[nm]	*R*_2_[nm]	*d*[nm]	T[K]	*τ*_exp_[s]	*τ*_SC_[s]	*τ*_VMC_[s]
Fe	20	12	30	750	30.0	7.5	3.3 × 10^7^
Fe	3.5	3.5	7	660	~0.8	1.8	2.7 × 10^7^
Fe§	~5.	~5.	~10.	725	~6.	2.1	2.7 × 10^7^
W^†^	20	20	100	1173	66.5	96.2	2.6 × 10^7^
W^†^	100	500*	100	1273	7.	8.6	1.5 × 10^5^

The self climb model is many orders of magnitude faster than VMC and retains accuracy over a wide range of loop sizes. Above: TEM observation of SIA loops in iron at 750 K, corresponding to the first entry in the above table. ^§^Data taken from Dudarev *et al*.^24^. ^†^Data taken from Ferroni *et al*.^9^. ^*^Pill shaped loop, length of ~50 nm, width ~20 nm.

## References

[b1] Hirth J. P. & Lothe J.. Theory Of Dislocations (Malabar, FL Krieger, 1991).

[b2] Mordehai D., Clouet E., Fivel M. & Verdier M.. Introducing dislocation climb by bulk diffusion in discrete dislocation dynamics. Philosophical Magazine 88, 899 (2008).

[b3] Bakó B., Clouet E., Dupuy L. M. & Blétry M.. Dislocation dynamics simulations with climb: kinetics of dislocation loop coarsening controlled by bulk diffusion. Philosophical Magazine 91, 3173 (2011).

[b4] Keralavarma S. M., Cagin T., Arsenlis A. & Benzerga A. A.. Power-Law Creep from Discrete Dislocation Dynamics. Physical Review Letters 109, 265504 (2012). 23368581 10.1103/PhysRevLett.109.265504

[b5] Maher D. M. & Eyre B. L.. Neutron irradiation damage in Molybdenum. Part V. Mechanisms of vacancy and interstitial loop growth during post-irradiation annealing. Philosophical Magazine 23, 409 (1971).

[b6] Silcox J. & Whelan M. J.. Direct observations of the annealing of prismatic dislocation loops and of climb of dislocations in quenched aluminium. Philosophical Magazine 5, 1 (1960).

[b7] Burton B. & Speight M. V.. The coarsening and annihilation kinetics of dislocation loops. Philosophical Magazine A 53, 385 (1986).

[b8] Yi X., Sand A. E., Mason D. R., Kirk M. A., Roberts S. G., Nordlund K. & Dudarev S. L.. Direct observation of size scaling and elastic interaction between nano-scale defects in collision cascades. Europhysics Letters 110, 36001 (2015).

[b9] Ferroni F., Yi X., Arakawa K., Fitzgerald S. P., Edmondson P. D. & Roberts S. G.. High temperature annealing of ion irradiated tungsten. Acta Materialia 90, 380 (2015).

[b10] Kroupa F. & Price P. B.. Conservative climb of a dislocation loop due to its interaction with an edge dislocation. Philosophical Magazine 6, 243 (1961).

[b11] Eyre B. L., Loretto M. H. & Smallman R. E.. Electron microscopy studies of point defect clusters in metals. Metal Science 12, 35 (1978).

[b12] Johnson C. A.. The growth of prismatic dislocation loops during annealing. Philosophical Magazine 5, 1255 (1960).

[b13] Turnbull J. A.. The coalescence of dislocation loops by self climb. Philosophical Magazine 21, 83 (1970).

[b14] Narayan J. & Washburn J.. Self climb of dislocation loops in magnesium oxide. Philosophical Magazine 26, 1179 (1972).

[b15] Khare S. V. & Einstein T. L.. Diffusion of monolayer adatom and vacancy clusters: Langevin analysis and Monte Carlo Simulations of their Brownian Motion. Physical Review B 54, 11752 (1996). 10.1103/PhysRevLett.75.2148 10059226

[b16] Sandberg N., Chang Z., Messina L., Olsson P. & Korzhavyi P.. Modeling of the magnetic free energy of self-diffusion in bcc Fe. Phys. Rev. B 92, 184102 (2015).

[b17] Bukonte L., Ahlgren T. & Heinola K.. Modelling of monovacancy diffusion in W over wide temperature range. Journal of Applied Physics 115, 123504 (2014).

[b18] Nguyen-Manh D., Horsfield A. P. & Dudarev S. L.. Self-interstitial atom defects in bcc transition metals: Group-specific trends. Physical Review B 73, 020101 (2006).

[b19] Sheng H. W., Kramer M. J., Cadien A., Fujita T. & Chen M. W.. Highly optimized embedded-atom-method potentials for fourteen fcc metals. Physical Review B 83, 134118 (2011).

[b20] Swinburne T. D. & Dudarev S. L.. Phonon drag force acting on a mobile crystal defect: Full treatment of discreteness and nonlinearity. Physical Review B 92, 134302 (2015).

[b21] Leetmaa M. & Skorodumova N. V.. KMCLib: A general framework for lattice kinetic Monte Carlo (KMC) simulations. Computer Physics Communications 185, 2340 (2014).

[b22] Mason D. R., Rudd R. E. & Sutton A. P.. Stochastic kinetic Monte Carlo algorithms for long-range Hamiltonians. Computer Physics Communications 160, 140 (2004).

[b23] Voter A. F.. Classically exact overlayer dynamics: Diffusion of rhodium clusters on Rh (100). Physical Review B 34, 6819 (1986). 10.1103/physrevb.34.6819 9939329

[b24] Dudarev S. L., Arakawa K., Yi X., Yao Z., Jenkins M., Gilbert M. R. & Derlet P. M.. Spatial ordering of nano-dislocation loops in ion-irradiated materials. Journal of Nuclear Materials 455, 16 (2014).

[b25] Arakawa K., Ono K., Isshiki M., Mimura K., Uchikoshi M. & Mori H.. Observation of the One-Dimensional Diffusion of Nanometer-Sized Dislocation Loops. Science 318, 956 (2007). 17991859 10.1126/science.1145386

[b26] Plimpton S.. Fast Parallel Algorithms for Short-Range Molecular Dynamics. Journal Computational Physics 117, 1 (1995).

[b27] Gordon P. A., Neeraj T. & Mendelev M. I.. Screw dislocation mobility in BCC Metals: a refined potential description for a-Fe. Philosophical Magazine 91, 3931 (2011).

[b28] Marinica M. C., Ventelon L., Gilbert M. R., Proville L., Dudarev S. L., Marian J., Bencteux G. & Willaime F.. Interatomic potentials for modelling radiation defects and dislocations in tungsten. Journal of Physics: Condensed Matter 25, 395502 (2013). 24002176 10.1088/0953-8984/25/39/395502

[b29] Henkelman G., Uberuaga B. P. & Jonsson H.. A climbing image nudged elastic band method for finding saddle points and minimum energy paths. The Journal of Chemical Physics 113, 9901 (2000).

[b30] Swinburne T. D., Dudarev S. L., Fitzgerald S. P., Gilbert M. R. & Sutton A. P.. Theory and simulation of the diffusion of kinks on dislocations in bcc metals. Physical Review B 87, 64108 (2013).

[b31] Derlet P. M., Gilbert M. R. & Dudarev S. L.. Langevin model for real-time Brownian dynamics of interacting nanodefects in irradiated metals. Physical Review B 84, 134109 (2011).

[b32] Dudarev S. L.. The non-Arrhenius migration of interstitial defects in bcc transition metals. Comptes Rendus Physique 9, 409 (2008).

[b33] Swinburne T. D., Dudarev S. L. & Sutton A. P.. Classical Mobility of Highly Mobile Crystal Defects. Physical Review Letters 113, 215501 (2014). 25479502 10.1103/PhysRevLett.113.215501

